# Effect of PCSK9 Inhibitor on Contrast-Induced Acute Kidney Injury in Patients with Acute Myocardial Infarction Undergoing Intervention Therapy

**DOI:** 10.1155/2022/1638209

**Published:** 2022-08-23

**Authors:** Yu Ma, Lei Zha, Qi Zhang, Lu Cao, Ru Zhao, Jing Ma, Kai Hou, Yue Pan, Hongliang Cong, Ximing Li

**Affiliations:** ^1^Department of Cardiology, Tianjin Chest Hospital, Tianjin 300222, China; ^2^Tianjin Institute of Cardiovascular Diseases, Tianjin Chest Hospital, Tianjin 300222, China; ^3^Tianjin Medical University, Tianjin Chest Hospital, Tianjin 300222, China; ^4^Department of Cardiology, Clinical School of Thoracic, Tianjin Medical University, Jinnan, Tianjin 300222, China

## Abstract

Proprotein convertase subtilisin/kexin type 9 (PCSK9) inhibitors have been shown to inhibit pyroptosis and apoptosis, which play important roles in the development and progression of contrast-induced acute kidney injury (CI-AKI). However, to the best of our knowledge, no studies have investigated the potential effect of PCSK9 inhibitors on the prevalence of CI-AKI after percutaneous coronary intervention (PCI). This study aimed to determine whether PCSK9 inhibitors are associated with the prevalence of CI-AKI. The medical records of 309 (mean age, 63.35 years; 71.84% male) patients with acute myocardial infarction who underwent PCI at our institution were retrospectively analyzed. Overall, 149 and 160 patients were assigned to the evolocumab and control groups, respectively. Serum creatinine levels were examined preoperatively and 24–72 h postoperatively and compared between groups. Data were grouped according to the occurrence of CI-AKI, and a univariate analysis was conducted to exclude suspected influencing factors that led to CI-AKI occurrence. After adjusting for confounding factors, a logistic regression analysis was performed to assess the association between evolocumab administration (independent variable) and CI-AKI occurrence (dependent variable). The prevalence of CI-AKI was significantly lower in the evolocumab group (6.7%) than in the control group (20.0%; *p* < 0.01).We further evaluated the correlation between exposure factor and outcome. The relative risk(RR) between the use of evolocumab and the occurrence of CI-AKI was 0.34(95% CI 0.17-0.66,p<0.01).This result indicate a significant association between the use of evolocumab and a reduction in the incidence of CI-AKI.The logistic regression analysis results revealed that evolocumab was significantly associated with CI-AKI. The use of PCSK9 inhibitors, hydration therapy, and statin administration appears promising for preventing CI-AKI in patients with acute myocardial infarction undergoing PCI.

## 1. Introduction

Coronary interventional procedures using intravascular contrast media are extensively performed worldwide. However, contrast-induced acute kidney injury (CI-AKI), a serious complication resulting from intravascular contrast media, occurs with an incidence of 10–15% during cardiac interventions and can reach 50% in high-risk populations [1]. Hydration therapy cannot prevent CI-AKI in high-risk patients [2]; therefore, identifying safe and effective methods to prevent CI-AKI is particularly important.

The Further Cardiovascular Outcomes Research with Proprotein Convertase Subtilisin/Kexin Type 9 (PCSK9) Inhibition in Patients with Elevated Risk (FOURIER) trial, Open-Label Study of Long-Term Evaluation against LDL-C (OSLER-1) trial, and Evolocumab for Early Reduction of LDL-Cholesterol Levels in Patients with Acute Coronary Syndromes (EVOPACS) study recommended PCSK9 inhibitors for the treatment of atherosclerotic cardiovascular disease [3–5]. As a type of PCSK9 inhibitor, evolocumab was administered on a large scale to treat acute coronary syndrome (ACS) [5]. PCSK9 inhibitors have been shown to inhibit pyroptosis and apoptosis [6–8], which play important roles in the development and progression of CI-AKI [9–11]. However, to the best of our knowledge, no studies have investigated the potential effect of PCSK9 inhibitors on the prevalence of CI-AKI after percutaneous coronary intervention (PCI).

This study aimed to investigate whether the administration of the PCSK9 inhibitor evolocumab combined with statin therapy in patients with acute myocardial infarction can reduce the prevalence of CI-AKI better than statin therapy alone.

## 2. Materials and Methods

### 2.1. Study Population

This study was a retrospective analysis of the medical files of 309 patients with acute myocardial infarction who underwent PCI at Tianjin Chest Hospital between January 2019 and December 2019. This study enrolled 222 male and 87 female patients with an average age of 63.35 (standard deviation (SD): 10.62) years.

The inclusion criteria for this study were as follows: male or female patients aged 18–90 years, ultra-high-risk atherosclerotic cardiovascular disease patients who met the diagnostic criteria for acute myocardial infarction, and patients receiving coronary intervention, aspirin combined with ticagrelor or clopidogrel, or treatment with atorvastatin or rosuvastatin, consistent with the indication for evolocumab.

We excluded 35 patients who met the following criteria: renal dysfunction, active renal disease or a need for renal replacement before the intervention, contraindications to the administration of PCSK9 inhibitors, active infection, malignancy, severe liver dysfunction, acute stroke, abnormal thyroid function, and Killip class >II cardiogenic shock.

The protocol of this study was approved by the Institutional Human Research Committee of Tianjin Chest Hospital Ethics Committee (approval no. 2021LW-004). This study was a retrospective review of medical records; therefore, the need for informed consent was waived.

### 2.2. Diagnostic Criteria for CI-AKI

CI-AKI was defined as *a* ≥25% or ≥44.2 *µ*mol/L increase in serum creatinine levels (compared with the baseline value) within 24–72 h of contrast medium administration [12]. This definition allowed the exclusion of patients with renal impairment induced by other causes.

### 2.3. Treatment Groups

First, the patients were assigned to groups according to whether or not the PCSK9 inhibitor evolocumab was administered before the intervention. There were 149 patients in the evolocumab group and 160 in the control group. The first group received 140 mg of evolocumab subcutaneously on admission to the emergency department. The control group did not receive evolocumab. The included patients were divided into 42 cases in the CI-AKI group and 267 cases in the non-CI-AKI group, according to the occurrence of CI-AKI postoperatively.

All patients were preoperatively treated with aspirin 300 mg, ticagrelor 180 mg or clopidogrel 600 mg, and atorvastatin 20 mg or rosuvastatin 10 mg. During the procedure, unfractionated heparin (80–100 U/kg) or bivalirudin (continued until 4 h after PCI) was administered. Furthermore, glycoprotein IIb/IIIa receptor antagonists, nicorandil, or sodium nitroprusside, were administered as indicated by the intraprocedural situation (slow flow or no-reflow). Most patients received the nonionic contrast agent iodixanol, while others received iopromide. Experienced interventional physicians in the Chest Pain Center at Tianjin Chest Hospital performed all PCI procedures. After PCI, all patients received standard medications for coronary artery disease, such as renin-angiotensin-aldosterone system (RAAS) inhibitors and beta-blockers, according to the guideline on the management of blood cholesterol, guideline for PCI, and guideline for the management of ST-elevation myocardial infarction, in addition to antiplatelet [13, 14], anticoagulation, and lipid-lowering therapies. If a patient with diabetes received metformin before the intervention, the administration of the drug was terminated 48 h postoperatively, and drug use was resumed only after a normal renal function review 48 h later. During this period, insulin was administered to control blood glucose levels as appropriate.

### 2.4. Baseline and Clinical Information on the Patients

Patient data recorded on admission included sex, age, body weight, previous myocardial infarction, history of hypertension and diabetes mellitus, and use of RAAS inhibitors, statins, or evolocumab.

### 2.5. Laboratory Parameters

#### 2.5.1. Blood Biochemistry

Serum creatinine, high-sensitivity C-reactive protein, high-density lipoprotein cholesterol, and low-density lipoprotein cholesterol (LDL-C) levels were measured using the Cobas c701 (Roche Diagnostics International AG, Rotkreuz, Switzerland) automatic biochemistry analyzer. Blood was collected from the cubital vein in all patients. Baseline serum creatinine levels and other indices were measured in patients after admission or as emergency checks before PCI. Serum creatinine indices were collected 24–72 h after the procedure. The remaining measurements were collected from patients after they fasted for 8 h the next morning.

#### 2.5.2. Cardiac Markers

Troponin T (TNT) and N-terminal pro-B-type natriuretic peptide (NT-proBNP) levels were measured using the Cobas e602 fully automated immunoassay analyzer (Roche Diagnostics International AG, Rotkreuz, Switzerland). Blood was collected from the cubital vein in all patients. Baseline TNT and NT-proBNP levels and other indices were measured in patients after admission or as emergency checks before PCI. The remaining measurements were collected from patients after they fasted for 8 h the next morning.

#### 2.5.3. Routine Blood Tests

The automated XN-9000 hematology analyzer (Sysmex Corporation, Kobe, Japan) was used for routine blood tests. Blood was collected from the cubital vein in all patients. Baseline routine blood tests were performed in patients after admission or as emergency checks before the intervention. The remaining measurements were collected from patients after they fasted for 8 h the next morning.

### 2.6. Echocardiography Indicators

All patients underwent examination with the CX50 cardiac ultrasound machine (Philips, Amsterdam, Netherlands).

### 2.7. Clinical Indicators

Clinical indicators included systolic and diastolic blood pressures taken on the day of the PCI, the intake of fluid during the first day after PCI, and the dose of the contrast agent.

### 2.8. Statistical Methods

Data analyses were performed using SPSS Statistics for Windows, version 25.0 (IBM Corp., Armonk, NY, USA). First, patients were assigned to groups according to whether or not the PCSK9 inhibitor evolocumab was administered before the intervention, and the change rate in serum creatinine level was compared between the two groups before and after the intervention. Second, the included patients were grouped according to whether or not they had CI-AKI after the intervention, and the suspected factors affecting CI-AKI were screened out. Continuous variables that conformed to a normal distribution were expressed as means and SDs, and the *t*-test was used to analyze such variables. Continuous variables that did not conform to the normal distribution were expressed as medians (P25–P75) and were analyzed using nonparametric tests.

Count data were expressed as percentages (%) and compared using the *χ*^2^ test. Logistic regression analysis was performed to assess whether the administration of evolocumab (independent variable) affected the occurrence of CI-AKI (dependent variable). According to the risk score for the prediction of CI-AKI [15], the model was adjusted for the following confounders: advanced age (>70 years), previous myocardial infarction, diabetes, hypertension, anemia (hemoglobin (Hb) level <110 g/L), contrast agent dose >150 mL, left ventricular ejection fraction (LVEF) <45%, emergency PCI, and suspicious influencing factors screened out by univariate analysis. A *p* value of <0.05 indicated statistical significance.

## 3. Results

### 3.1. Comparison of Baseline Data between the Evolocumab and Control Groups

The age of the patients included in this study varied from 27 to 90 (63.35, SD: 10.62) years, and the percentage of male patients was 71.84%. The percentage of iodixanol use was significantly lower in the evolocumab group than in the control group (*p* < 0.05). No significant difference was observed regarding all other parameters between the groups (*p* > 0.05) (Table 1).

### 3.2. Comparison of CI-AKI Indicators between the Evolocumab and Control Groups

The difference in the post-PCI serum creatinine levels between the evolocumab and control groups was significant (*Z* = −3.69, *p* < 0.01), and the creatinine level in the control group was higher than that in the evolocumab group. The changes in serum creatinine levels from pre-PCI to post-PCI were significantly different between the two groups (*Z* = −3.28, *p* < 0.01). The prevalence of CI-AKI was significantly higher (*χ*^2^ = 11.6, *p* < 0.01) in the control group than in the evolocumab group (Table 2).We further evaluated the correlation between exposure factor and outcome. The relative risk(RR) between the use of evolocumab and the occurrence of CI-AKI was 0.34(95% CI 0.17-0.66,p<0.01).This result indicate a significant association between the use of evolocumab and a reduction in the incidence of CI-AKI.

### 3.3. Comparison of Baseline Data between the CI-AKI and Non-CI-AKI Groups

On re-examination of serum creatinine levels, 42 of the 309 patients met the diagnostic criteria of CI-AKI, and the incidence of CI-AKI was 13.59%. Univariate analysis revealed significant differences in diabetes history, Killip class I, and use of evolocumab (all *p* < 0.05) between the CI-AKI and non-CI-AKI groups (Table 3).

### 3.4. Association between Evolocumab and CI-AKI Prevalence

**Figure 1:**
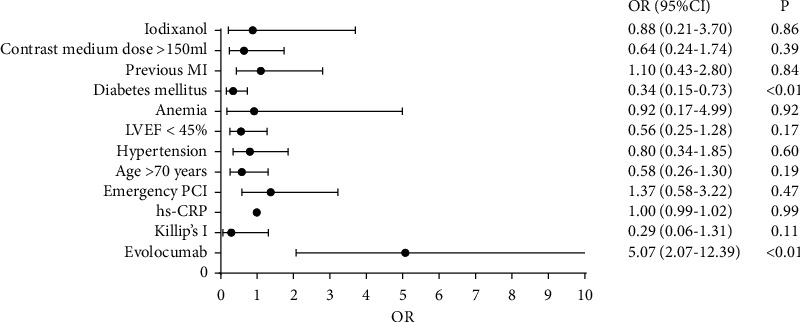
Logistic regression analysis of patients with acute myocardial infarction undergoing coronary intervention (*n* = 309). OR, odds ratio; CI, confidence interval; hs-CRP, high-sensitivity C-reactive protein; PCI, percutaneous coronary intervention; LVEF, left ventricular ejection fraction; MI, myocardial infarction.

Three suspected risk factors for CI-AKI identified using univariate analysis were diabetes mellitus, evolocumab use, and Killip class I. Regression analysis was performed on these three factors and other clinically recognized factors affecting CI-AKI occurrence [15], including advanced age (>70 years), previous myocardial infarction, diabetes, hypertension, anemia (Hb level <110 g/L), contrast agent dose >150 mL, LVEF <45%, and emergency PCI. The results showed that the use of evolocumab was a protective factor for CI-AKI occurrence (odds ratio (OR) = 0.28, 95% CI 0.11–0.71, *p* < 0.01), whereas diabetes mellitus and LVEF <45% were risk factors for CI-AKI (Table 4, Figure 1).

## 4. Discussion

This retrospective study investigated the prevalence of CI-AKI after PCI, the preferred treatment for acute myocardial infarction. Our results show that patients who received evolocumab, a PCSK9 inhibitor, were significantly less likely to have CI-AKI than those who did not receive evolocumab.

The prevention and treatment of iatrogenic complications have become important topics in healthcare. The currently used CI-AKI prevention measures included reducing the dose of contrast agents, using isotonic or hypotonic nonionic contrast agents, and sufficiently hydrating patients.

In this study, the proportion of patients who received isotonic contrast agent (iodixanol) in the control and evolocumab groups was 96.25% and 79.19%, respectively (*p* < 0.01), grouped according to the presence or absence of evolocumab. The proportion of patients who received iodixanol in the non-CI-AKI and CI-AKI groups was 87.27% and 92.86%, respectively (*p*=0.30), grouped according to the presence or absence of CI-AKI. The proportion of patients who received hypotonic contrast in the control and evolocumab group was 3.75% and 20.81%, respectively. Theoretically, the incidence of CI-AKI should be higher in the evolocumab group than in the control group. However, the actual results showed that the opposite was true, such that the incidence of CI-AKI was lower in the evolocumab group, suggesting that PCSK9 inhibitors protected against CI-AKI. Therefore, we believe that the application of PCSK9 inhibitors may be a protective factor in the development of CI-AKI.

Hydration therapy administered orally or intravenously is currently recognized as the most effective means of CI-AKI prevention. Hydration during the peri-PCI period dilutes the contrast agent as it enters the body, reducing the effects of its osmotic pressure and concentration on the kidneys. However, hydration therapy cannot prevent CI-AKI in high-risk patients, and approximately 11% of patients with chronic renal dysfunction develop CI-AKI despite adequate hydration therapy [2].

Recently, the role of statins in preventing CI-AKI has been investigated more extensively. Statins improve endothelial function, inhibit inflammation and apoptosis, and exert antioxidant and antithrombotic effects [16]. A retrospective analysis by Khanal et al. showed that statin administration before PCI significantly reduced CI-AKI incidence [17]. However, less than one-third of Chinese patients who receive statin therapy comply with medication adherence, especially those with very high risks of atherosclerotic cardiovascular disease [18]. Simply optimizing statin therapy cannot meet the demand for lipid reduction, and more intensive therapy is needed. The American Society of Nephrology guidelines emphasize stricter blood lipid management for patients with cardiovascular and chronic kidney diseases [19]. The addition of evolocumab to statin therapy has further reduced LDL-C levels by 59–75% [3, 4, 20]. The EVOPACS study showed that evolocumab administration within 1–3 days of the commencement of an ACS allowed >95% of patients to achieve an LDL-C level of <1.8 mmol/L [5].

In pre-PCI statin and hydration therapy, the results showed that the prevalence of CI-AKI in patients with acute myocardial infarction who received evolocumab was 6.7%, which was significantly less than the rate of 20.0% in the group that did not receive the PCSK9 inhibitor. Logistic regression analysis confirmed that the PCSK9 inhibitor was a protective factor against CI-AKI in patients undergoing PCI. Therefore, this study explored whether PCSK9 inhibitors reduce the prevalence of CI-AKI in patients undergoing PCI.

The mechanism underlying this reduction in CI-AKI prevalence after PCI in patients receiving evolocumab is not entirely clear, although there are a few potential explanations. A meta-analysis concluded that PCSK9 inhibitors inhibit pyroptosis independent of their lipid-lowering effects [6]. A study showed that PCSK9 regulates pyroptosis through mitochondrial DNA damage in mice with chronic myocardial ischemia [21]. Pyroptosis is a form of programmed cell death observed in many types of cells, including immune cells, vascular smooth muscle cells, cardiomyocytes, and renal tubular epithelial cells [22]. PCSK9 was closely associated with macrophage pyroptosis and the progression of inflammation in atherosclerosis. Caspase-11-mediated tubular epithelial pyroptosis has been confirmed as the basis of CI-AKI [23]. Consequently, reducing pyroptosis may diminish the degree of contrast-induced tubular epithelial injury.

Some studies have shown that reduced PCSK9 transcription is associated with increased expression of the apolipoprotein E2 receptor. Cells lacking this receptor gene lose their PCSK9-dependent apoptosis ability, whereas the inhibition of PCSK9 expression may also reduce apoptosis [7, 8]. Contrast-induced apoptosis in vascular endothelial and tubular epithelial cells promotes CI-AKI development [10, 11]. Based on these findings, it is hypothesized that the administration of PCSK9 inhibitors may reduce CI-AKI by decreasing pyroptosis and apoptosis.

With the growing evidence on the effects of PCSK9 inhibitors, their importance in treatment guidelines has increased significantly. The Fourier and OSLER-1 trials and EVOPACS study recommend PCSK9 inhibitors for treating atherosclerotic cardiovascular disease [3–5]. PCSK9 inhibitors are a new type of lipid-lowering drug with a more robust lipid-lowering effect and higher safety profile.

The results of this study should be interpreted within its limitations. First, this was a nonrandomized retrospective study whose inherent weakness cannot be avoided. Second, this study was conducted in a single center; therefore, it may be weaker in methodology than studies using multicenter sampled populations. Third, the effects of many drugs have not been ruled out, especially some renal protective drugs.

## 5. Conclusions

This study shows that PCSK9 inhibitor therapy combined with hydration and statin administration is a promising method to prevent CI-AKI. The sample size of this single-center clinical retrospective study was limited because of which it was impossible to exclude the influence of confounding factors completely. Moreover, this study only investigated the incidence of CI-AKI in patients with acute myocardial infarction; the effect of PCSK9 inhibitors on CI-AKI incidence in other ASCVD (Atherosclerotic Cardiovascular Disease) patients undergoing PCI remains to be prospectively studied.

## Figures and Tables

**Table 1:** Comparison of baseline data between those who received evolocumab and controls.

	Control group (*n* = 160)	Evolocumab group (*n* = 149)	*χ* ^2^/*t*/*Z*	*p* value
Male sex, *n* (%)	120 (75.0)	102 (68.46)	1.63	0.20
Age (years), mean, SD	64.15, 10.73	62.49, 10.45	1.38	0.17
STEMI, *n* (%)	103 (64.38)	99 (66.44)	0.15	0.70
Old MI, *n* (%)	28 (17.50)	26 (17.45)	<0.01	0.99
Diabetes mellitus, *n* (%)	58 (36.25)	57 (38.26)	0.13	0.72
Hypertension, *n* (%)	88 (55.00)	96 (64.43)	2.85	0.09
SBP (mmHg), median (IQR)	134.00 (123.00–145.00)	134.00 (123.00–145.00)	−0.14	0.89
DBP (mmHg), median (IQR)	78.00 (70.00–89.00)	78.00 (70.00–88.75)	−0.60	0.55
Atorvastatin, *n* (%)	91 (56.88)	80 (53.69)	0.32	0.57
RAAS inhibitors, *n* (%)	143 (89.38)	126 (84.56)	1.59	0.21
Killip class I, *n* (%)	157 (98.13)	141 (94.63)	2.74	0.10
LVEF (%), median (IQR)	50.00 (45.00–56.00)	51.50 (46.00–56.00)	−0.87	0.39
NT-pro-BNP (ng/L), median (IQR)	684.00 (206.20–1582.00)	719.60 (328.65–1510.00)	−0.57	0.57
TNT (ng/mL), median (IQR)	1.74 (0.64–3.39)	1.43 (0.43–3.11)	−1.43	0.15
Hb (g/L), median (IQR)	133.00 (125.00–145.00)	134.00 (122.25–145.00)	−0.01	0.99
Hs-CRP (mg/L), median (IQR)	6.34 (2.25–23.10)	5.01 (2.13–13.67)	−1.16	0.25
HDL-C (mmol/L), median (IQR)	1.03 (0.87–1.24)	1.00 (0.83–1.20)	−1.11	0.27
LDL-C (mmol/L), median (IQR)	3.17 (2.51–3.87)	3.23 (2.43–4.24)	−0.02	0.99
Contrast agent (mL), median (IQR)	140.00 (130.00–150.00)	140.00 (120.00–150.00)	−0.73	0.47
Emergency PCI, *n* (%)	103 (64.38)	99 (66.44)	0.15	0.70
Preoperative hydration, *n* (%)	43 (26.88)	29 (19.46)	2.37	0.12
Postoperative hydration, *n* (%)	157 (98.13)	147 (98.66)	0.14	0.71
Iodixanol, *n* (%)	154 (96.25)	118 (79.19)	21.29	<0.01

*χ* ^2^/*t*/*Z*, a test used to compare parameters; SD, standard deviation; STEMI, acute ST-segment elevation myocardial infarction; MI, myocardial infarction; SBP, systolic blood pressure; IQR, interquartile range; DBP, diastolic blood pressure; RAAS, renin-angiotensin-aldosterone system; LVEF, left ventricular ejection fraction; NT-pro-BNP, N-terminal probrain natriuretic peptide; TNT, troponin T; Hb, hemoglobin; hs-CRP, high-sensitivity C-reactive protein; HDL-C, high-density lipoprotein cholesterol; LDL-C, low-density lipoprotein cholesterol; PCI, percutaneous coronary intervention.

**Table 2:** Comparison of pre- and postintervention kidney function and contrast-induced acute renal injury between those who received evolocumab and controls.

	Control group (*n* = 160)	Evolocumab group (*n* = 149)	*χ* ^2^/*t*/*Z*	*p* value
Pre-PCI Cr (*μ*mol/L), median (IQR)	78.00 (67.00–89.00)	78.00 (65.00–87.5)	−0.96	0.34
Post-PCI Cr (*μ*mol/L), median (IQR)	86.00 (73.00–99.00)	80.00 (69.00–90.00)	−3.69	<0.01
Cr change pre- to post-PCI (%), median (IQR)	7.14 (1.12–20.26)	3.45 (−1.18–12.79)	−3.28	<0.01
CI-AKI, *n* (%)	32 (20.00)	10 (6.70)	11.60	<0.01

*χ* ^2^/*t*/*Z*, a test used to compare parameters. IQR, interquartile range; PCI, percutaneous coronary intervention; Cr, creatinine; CI-AKI, contrast-induced acute renal injury.

**Table 3:** Comparison of baseline data between the CI-AKI and non-CI-AKI groups.

	Non-CI-AKI group (*n* = 267)	CI-AKI group (*n* = 42)	*χ* ^2^/*t*/*Z*	*p* value
Male sex, *n* (%)	194 (72.66)	28 (66.67)	0.64	0.42
Age (years), mean, SD	63.07, 10.78	65.14, 9.45	−1.18	0.24
STEMI, *n* (%)	173 (64.79)	29 (69.05)	0.29	0.59
Old MI, *n* (%)	45 (16.85)	9 (21.43)	0.53	0.47
Diabetes mellitus, *n* (%)	90 (50.85)	25 (59.52)	10.35	<0.01
Hypertension, *n* (%)	154 (57.68)	30 (71.43)	2.85	0.09
SBP (mmHg), median (IQR)	134.00 (123.00–145.00)	135.00 (122.50–150.50)	−1.00	0.32
DBP (mmHg), median (IQR)	78.00 (70.00–86.00)	80.00 (69.75–90.50)	−0.73	0.47
Evolocumab, *n* (%)	139 (52.06)	10 (23.81)	11.60	<0.01
Atorvastatin, *n* (%)	147 (55.06)	24 (57.14)	0.06	0.80
RAAS inhibitors, *n* (%)	229 (85.77)	40 (95.24)	2.89	0.09
Killip class I, *n* (%)	260 (97.38)	38 (90.48)	5.04	0.03
LVEF (%), median (IQR)	51.00 (46.00–56.00)	50.00 (42.00–56.25)	−1.11	0.27
NT-pro-BNP (ng/L), median (IQR)	679.00 (212.50–1475.00)	852.50 (428.00–2489.00)	−1.56	0.12
TNT (ng/mL), median (IQR)	1.59 (0.54–3.19)	1.50 (0.55–6.67)	−0.87	0.39
Hb (g/L), median (IQR)	133.00 (123.00–144.00)	137.00 (126.00–148.00)	−1.30	0.20
Hs-CRP (mg/L), median (IQR)	5.19 (2.10–18.60)	9.35 (2.64–15.63)	−1.18	0.24
HDL-C (mmol/L), median (IQR)	1.01 (0.86–1.23)	1.05 (0.82–1.35)	−0.56	0.58
LDL-C (mmol/L), median (IQR)	3.17 (2.46–4.21)	3.35 (2.57–3.75)	−0.54	0.59
Contrast agent (mL), median (IQR)	140.00 (130.00–150.00)	140.00 (130.00–150.00)	0.65	0.52
Emergency PCI, *n* (%)	171 (64.04)	31 (73.81)	1.53	0.22
Preoperative hydration, *n* (%)	60 (28.99)	12 (28.57)	0.76	0.39
Postoperative hydration, *n* (%)	263 (98.50)	41 (97.62)	0.18	0.67
Iodixanol, *n* (%)	233 (87.27)	39 (92.86)	1.08	0.30

*χ* ^2^/*t*/*Z*, a test used to compare parameters. CI-AKI, contrast-induced acute renal injury; SD, standard deviation; STEMI, ST-segment elevation myocardial infarction; MI, myocardial infarction; SBP, systolic blood pressure; IQR, interquartile range; DBP, diastolic blood pressure; RAAS, renin-angiotensin-aldosterone system; LVEF, left ventricular ejection fraction; NT-pro-BNP, N-terminal pro-brain natriuretic peptide; TNT, troponin T; Hb, hemoglobin; hs-CRP, high-sensitivity C-reactive protein; HDL-C, high-density lipoprotein cholesterol; LDL-C, low-density lipoprotein cholesterol; PCI, percutaneous coronary intervention.

**Table 4:** Logistic regression analysis of patients with acute myocardial infarction undergoing coronary intervention (*n* = 309).

	*B*	SE	Wald *c*^2^	*p* value	OR	95% CI
Evolocumab	1.62	0.46	12.65	<0.01	5.07	2.07–12.39
Killip class I	−1.26	0.78	2.59	0.11	0.29	0.06–1.31
Hs-CRP	<0.01	0.01	<0.01	0.99	1.00	0.99–1.02
Emergency PCI	0.31	0.44	0.51	0.47	1.37	0.58–3.22
Age >70 years	-0.55	0.42	1.76	0.19	0.58	0.26–1.30
Hypertension	−0.23	0.44	0.28	0.60	0.80	0.34–1.85
LVEF <45%	−0.58	0.42	1.87	0.17	0.56	0.25–1.28
Anemia	−0.09	0.87	0.01	0.92	0.92	0.17–4.99
Diabetes mellitus	−1.09	0.40	7.50	<0.01	0.34	0.15–0.73
Previous MI	0.10	0.48	0.04	0.84	1.10	0.43–2.80
Contrast medium dose >150 mL	−0.44	0.51	0.76	0.39	0.64	0.24–1.74
Iodixanol	−0.13	0.73	0.03	0.86	0.88	0.21–3.70

*B*, beta; SE, standard error; OR, odds ratio; CI, confidence interval; hs-CRP, high-sensitivity C-reactive protein; PCI, percutaneous coronary intervention; LVEF, left ventricular ejection fraction; MI, myocardial infarction.

## Data Availability

All data generated or analyzed during this study are available from the corresponding author on reasonable request.
